# Food allergy issues among consumers: a comprehensive review

**DOI:** 10.3389/fnut.2024.1380056

**Published:** 2024-03-26

**Authors:** Samantha Sansweet, Ria Jindal, Ruchi Gupta

**Affiliations:** ^1^Center for Food Allergy and Asthma Research, Feinberg School of Medicine, Northwestern University, Chicago, IL, United States; ^2^Ann and Robert H. Lurie Children’s Hospital of Chicago, Chicago, IL, United States

**Keywords:** food allergy, quality of life, psychosocial burden, public health, social determinants of health

## Abstract

Food Allergy (FA) is a growing global public health concern. In the United States alone, 8% of children and 11% of adults have a convincing FA (symptoms consistent with an IgE-mediated reaction to a specific allergen). Given the significant prevalence of this condition, the objective of this mini-review is to illustrate the many dimensions of life that are impacted among those with FA. Summarizing findings from a breadth of current literature, we present how FA affects social, psychological, and economic-related quality of life. With this informative review, we endeavor to bring increased awareness to these issues and help cultivate a better future for individuals with FA.

## 1 Introduction

Food allergy (FA) is a significant public health concern, impacting about 8% of children and 11% of adults in the United States (1, 2). Although you can be allergic to any food, the top nine food allergens are peanuts, milk, shellfish, tree nuts, eggs, finfish, wheat, soy, and sesame. Every allergic reaction is unique to the individual and the situation, with 42% of children experiencing severe reactions reporting at least one lifetime visit to the Emergency Department (ED) (1). Although FA can develop at any age, certain allergens may be more prevalent at certain stages of life. In early life, milk is the most common allergen, impacting 53% of children <1 year old; in adolescence, peanuts are the most common impacting 29.5% of children >14 years old; and once individuals reach adulthood, shellfish is the most common, impacting 2.9% of adults (1). Children with FA are also significantly more likely to have atopic comorbidities, including asthma, atopic dermatitis, eosinophilic esophagitis (EoE), and allergic rhinitis. When comparing prevalence for all children to those with FA, the following were shown: for asthma, 12.2 to 32.6%; for atopic dermatitis, 5.9 to 14.9%; for EoE, 0.2 to 0.7%; for allergic rhinitis, 12.8 to 30.4% (1). Throughout this mini-review, we will explore the psychosocial and economic impacts on FA-related quality of life (QoL).

## 2 Daily quality of life with food allergy

The CDC defines health-related QoL as “an individual’s or group’s self-perception of their physical and mental health over time” (CDC-HRQOL) (3). Living with FA impacts both of these aspects and although there are many emerging treatments that may help mitigate the severity and reduce the burden, there is no cure. Food allergic individuals and their families must adapt and learn how to navigate the world with a potentially life-threatening food condition. One study assessing the psychosocial burden of FA among US adults found that multiple FA, severe reactions, a current epinephrine auto-injector prescription, a history of epinephrine use for FA treatment, and a FA-related ED visit in the last 12 months are all factors indicative of a higher Food Allergy Independent Measure (FAIM) score (4). The FAIM score assesses an individual’s “perceived risk of accidental allergen exposure and the severity of the anticipated outcome” (4). Therefore, a higher FAIM score indicates a greater impact on QoL. The daily burden of FA management can increase stress and anxiety surrounding the fear of having a reaction, the ability to treat said reaction, and even the possibility of FA-related bullying. This need for hypervigilance can lead to avoidance, social isolation, and decreased QoL (5, 6). Areas of life where QoL impacts may be most abundant include the school setting, social interactions, family relationships, finances, allergen labeling on packaged foods, shopping for safe foods, dining out, and traveling.

### 2.1 School-related impacts

School is a place for children to learn and grow academically and socially. In early childcare settings, it is important to recognize that since children often cannot advocate for themselves, parents place their trust in the school to protect them and respond appropriately to a FA emergency. A survey of early childcare professionals reported 38% felt unprepared to administer epinephrine if a severe allergic reaction were to occur, more than 25% were unfamiliar with an Emergency Action Plan (EAP; outlines the steps to take when a specific child is having an allergic reaction), and less than half of respondents reported being comfortable identifying allergy-friendly food labels (7). Increasing efforts must be made to better prepare early childcare professionals to communicate with nonverbal children, recognize the signs of an allergic reaction in a young child, and know how to respond effectively.

About 18% of children with FA have had an allergic reaction at school and 25% experienced an anaphylactic reaction for the first time at school (8). In elementary and middle school, children may experience new stressors among their peers. Children without FA often do not understand their severity and how dangerous they can be. As a result, there is an opportunity for bullying to occur. One study found that 57% of participants reported they were either touched with their allergen, it was thrown at them, or their food was purposefully contaminated with the allergen (9). These interactions can further exacerbate the fear and anxiety associated with food allergies for the child and their caregiver. The child may feel unsafe attending school and caregivers may fear for their child’s safety. Additional consequences to QoL could include the child missing school due to a visit to the ED, fear and anxiety leading to increased isolation (i.e., sitting separately from peers at lunch and, feeling different from one’s peers) and the child’s caregiver missing work.

As a child moves into adolescence and young adulthood, FA can lead to increased stress and risk-taking behaviors. An important indicator of engagement in risk-taking behaviors is the individual’s support system. With less support, individuals are less likely to carry an epinephrine auto-injector, speak up at a restaurant, discuss their FA in social settings, and check food labels when trying foods (10). Adolescence and young adulthood is a time for individuals to discover themselves and find where they fit in, which can be difficult when they feel different from their peers. FA management requires hypervigilance and preparedness to avoid an emergency. While others may not think twice about going to a restaurant, for example, it can be isolating to be the odd one out, who may opt not to eat the food or not go in the first place.

The transition to college can be a daunting process for students with FA as they leave the support system they have built at home and independently manage their FA for the first time. One study gathered insight from college students and their parent’s experiences, and many expressed concerns for their safety regularly and the detrimental toll it takes on their mental health. These concerns stemmed from dining halls being ill-prepared to safely prepare and label food correctly, roommates being careless with allergens, the fear of kissing someone who had previously eaten the allergen, and the fear of having a reaction and having to miss school which can negatively impact their studies (11). It is increasingly important to educate everyone on the severity of FA, implore them to take FA seriously, and implement safeguards and policies that will protect students and create an environment that allows for peace of mind and the ability to focus on academics without fear.

### 2.2 Work-related impacts

There is very limited research formally investigating the impacts of managing FA in the workplace, but there are countless articles, blogs, and podcasts of firsthand experiences and advice available. One article through the *Harvard Business Review* explored workplace inclusivity regarding FA, stating that “one in three people with FA report feeling uncomfortable or unsafe at work, and 60% of millennials with FA report experiencing anxiety at work because of their condition” (12). This is consistent with previous research concluding that FA adults’ QoL is often impaired, and they may experience increased stress and anxiety (4, 13). It can be challenging to navigate the workplace as a FA adult due to concerns over being a burden, fear of cross-contact if proper precautions are not taken, and feeling isolated as most work events involve food (12). There is also the concern of FA bullying which persists through adulthood. Michele Payn’s book and subsequent podcast on food bullying recounts an anonymous account of bullying in the workplace. The individual described coworkers intentionally leaving out open bowls of peanuts during meetings and at cubicles, while being aware of their severe peanut allergy (14). The burden of FA persists through adulthood and can greatly impact work life. Fortunately, FA is covered under The Americans with Disabilities Act of 1990 (ADA), therefore there are legal protections in place to combat FA-related discrimination (15). FA adults can advocate for change and bring awareness to employers and co-workers by talking with management, suggesting social events are non-food related, and sharing educational resources on food allergies, such as Food Allergy Research and Education’s (FARE) series on food allergies in the workplace (16).

### 2.3 Social interactions and relationships

As humans, our QoL is deeply tied to social interactions and relationships. One significant way people connect is through food, which is a major part of our identity and culture. Food allergies can put a strain on many people and relationships as their impacts are ingrained in our everyday lives. We have food at almost every social gathering, i.e., parties, sporting events, dining out, work conferences, etc. Research has found that many families with a FA child will often eat at the same restaurants they know are safe, avoid eating out completely, and limit travel and vacations if they must fly or stay overnight (17). In addition, the added burden has been found to cause strain on marriages, limit the child’s ability to play at a friend’s house or participate in parties, sports, and camps, and even cause caregivers to adjust their work schedules or stop working altogether to homeschool their children (5). One study exploring personality traits and FA experiences found that extroverted FA adults report more social challenges attributed to their FA, “such as people being unkind toward them and feeling anxious or stressed in social occasions and lack of understanding from others (18). Food allergies can be managed, but the burden can often be overwhelming when people do not feel they have adequate support to ensure their safety in food-related situations.

### 2.4 Food labeling practices

Navigating food labels and shopping for allergen-safe foods can be a daunting task. There is little regulation on food labels, specifically when it comes to precautionary allergen labeling (PAL) such as “may contain” or “manufactured on shared equipment” statements. Shoppers should always check ingredient labels in case of ingredient changes, or the addition of new allergens. Fortunately, the Food Allergen Labeling and Consumer Protection Act (FALCPA) requires labeling major allergens on packaged foods (19). According to a study assessing people’s understanding of PALs, over half of those surveyed reported current labeling practices interfering with their daily lives and expressed interest in more information on the meaning behind PALs. In addition, 27% of respondents reported themselves or a family member having an allergic reaction after eating a food item with a PAL statement (20). Clear policies must be implemented to ensure clarity and consistency of PALs and the safety of FA consumers.

### 2.5 Economic impact and disparities

The financial burden of food allergy is another significant factor that can impact people’s lives. The overall economic cost of FA is 24.8 billion, with 4.8 billion attributed to direct medical costs and 20.5 billion to family costs (21). In a cross-sectional survey given to 1,643 caregivers of children with current food allergies, the most frequent and common costs were hospitalizations, ED visits, special diets and allergen-free food, changes in childcare, special summer camps, changing schools due to FA, and missed work or job loss among caregivers (21). Unfortunately, managing FA and providing safe experiences comparable to those without FA can be incredibly expensive.

In addition, socioeconomic disparities that can increase the financial burden and negatively impact one’s QoL must be considered. Research has found that historically underrepresented population groups using Medicaid often utilize the ED more frequently and are less likely to be diagnosed with a FA due to difficulty accessing specialized care through an allergist (22). Ensuring all FA patients have access to affordable specialty care, such as an allergist or primary care physician must be prioritized. Other factors to consider may include living in a food desert (areas with limited access to affordable and nutritious food) or food swamp (areas where fast food and junk food are more prevalent than healthier options) and experiencing food insecurity, which can further limit the availability and affordability of allergen-safe foods and negatively impact the nutritional quality of the individual’s diet if the less nutritious option is deemed safer, more convenient, and more affordable (23, 24). These added burdens and lack of needed resources can further increase the stress and anxiety associated with managing a FA. Therefore, systems must be in place to support these individuals and families, such as routine food insecurity screening among FA patients (25).

## 3 Discussion

Managing FA is more complex than simply avoiding the allergen (s). The burden of the disease is on the individual and their entire support system, including family, friends, and the surrounding community, and can impact all aspects of life. In a study examining mothers’ experiences of raising a child with FA, participants reported encountering skepticism about the severity of their child’s allergy, judgment about their management approach, and expressed concerns about balancing politeness with ensuring their child’s safety during food-related situations (26). It is easy to dismiss the severity of this burden if you are not directly affected, but by bringing awareness to the QoL impacts we can create safe, inclusive, and supportive communities for those struggling to manage FA.

There are several possible solutions to increasing awareness and positively impacting QoL, as outlined in Table 1. The first is to be intentional about creating a supportive community, which could look different for each person. One study found that 62% of early childcare professionals did not understand the terminology often used in an EAP (7). A helpful way to support your child would be through outlining an EAP with your child and their doctor and taking the time to sit down with their teachers and explain all the needed steps. It is also important for parents to empower their children and help them see the positives associated with their FA. Adolescents and young adults have reported that living with their FA has made them feel more responsible and helped them to better advocate for themselves and others (10). Parents should also seek counseling and/or support groups for themselves and their child to help manage and navigate the added stress and anxiety and share experiences with families in similar situations (5).

**Table 1:** Summary of food allergy-related impacts on consumers and proposed solutions.

Quality of life impacts	Proposed solutions
**School-related** Safety, fear, anxiety, bullying, isolation, missed school/work Lack of teacher preparedness (i.e., food labels, epinephrine autoinjector administration) Risk-taking behaviors in adolescence and young adulthood *See *School-Related Impacts & Discussion* Sections	Education and training Teachers, nurses, staff, and students Epinephrine use, signs and symptoms, cross-contact, safe-cleaning practices, reading food labels Emergency Action Plan Support system Advocate for self and/or child
**Work-related** Safety, fear, anxiety, bullying, isolation, missed work Lack of knowledge and awareness among co-workers/employer Work events (i.e., dinners, conferences) *See *Work-Related Impacts* Section	Education and training All staff Sharing resources Epinephrine use, signs and symptoms, cross-contact, safe-cleaning practices, reading food labels Advocate for self FA is protected under the ADA
**Social Interactions & Relationships** Food is a part of daily life (parties, restaurants, traveling, playdates, camps, etc.) Marriage strain on caregivers of FA child Lack of awareness and understanding *See *Social Interactions and Relationships & Discussion* Sections	Peer and family education Sharing resources Support system Counseling, support groups Peer support Advocating for self/child Safe dating practices
**Allergen Labeling** Minimal regulation on PALs Vigilance in checking labels in case of changes *See *Food Labeling Practices & Discussion* Sections	Advocate for policy changes FALCPA (19) FASTER Act
**Economic Burden and Health Disparities** $24.8 billion overall $4.8 billion in direct medical costs $20.5 billion in family costs Hospitalizations, ED visits, special diets and allergen-free food, changes in childcare, special summer camps, changing schools due to FA, and missed work or job loss among caregivers Financial burden Food insecurity Lack of access to care *See *Economic Burden and Health Disparities & Discussion* Sections	Expanding insurance coverage Volunteer allergists at clinics Advocate for stock-epinephrine in schools and public places Build a more diverse workforce more likely to advocate for change

Food allergy education is vital to increasing community awareness and preparedness in case of a FA emergency. A review by Sansweet et al. (27) suggests that many negative QoL impacts of FA may stem from a lack of knowledge and awareness. This review highlights the importance of prioritizing community-based education initiatives on reaching high risk communities with limited access to resources. Public health workers should encourage schools, workplaces, and restaurants to provide regular education and training on FA. Education is a simple and effective way to increase awareness and improve knowledge and perceptions of FA. Teachers are often ill-equipped to manage a FA emergency even though they are often the first line of defense for students having a reaction. Training has been shown to increase awareness and teacher’s confidence and ability to respond effectively in a FA emergency (28). The implementation of frequent and recurrent training can aid in increasing teachers’ ability to correctly manage using epinephrine autoinjectors. In addition, students can also benefit from receiving FA education as their lack of knowledge can often lead to bullying situations that can become dangerous and life-threatening. Discussions should be encouraged at school and at home. Helpful education resources for all ages can be found on the Center for Food Allergy and Asthma Research website to help facilitate conversation (29).

Advocacy can be an effective tool for affecting policy change and has already proven useful at the federal level. Advocates have worked to regulate PALs across the US for many years. The Food Allergen Labeling and Consumer Protection Act (FALCPA) (19) became law in 2004, and that was the last time any changes were made since 2021 when the Food Allergy Safety, Treatment, Education, and Research (FASTER) Act was implemented. This law requires the addition of sesame along with the other previous top allergens to be labeled on packaged foods sold in the US and went into effect on January 1^st^, 2023 (30). This is an exciting and welcome addition that will help create peace of mind for shoppers allergic to sesame.

Lastly, medical professionals, researchers, advocacy groups, and policy makers must work together to mitigate the financial burden placed on families and reduce health disparities. There is not just one solution, but many systems that need to work together to ensure affordable and accessible care for everyone. This may include expanding insurance coverage, having allergists volunteer their time at clinics in underserved communities, or advocating for stock epinephrine in schools and public spaces. In addition, building a more diverse workforce of healthcare professionals who are more likely to advocate for change can build trust among patients of similar backgrounds and cultivate a more welcoming and accessible healthcare experience (31).

The public health burden of FA is complex and multifaceted. We must look at the issue from a holistic lens and ensure we capture the entire experience and acknowledge the toll it can take on one’s physical and mental health. Hypervigilance is needed to ensure one’s safety. In addition, the fear and anxiety of a potential reaction are often compounded by the social and financial pressures of being able to advocate for oneself among peers and afford safe foods and needed treatment. Increasing awareness of both the severity and QoL impacts of living with and managing the daily stressors associated with having a FA are imperative for creating a community of understanding and support.

## Author contributions

SS: Writing – original draft, Writing – review & editing. RJ: Writing – original draft, Writing – review & editing. RG: Writing – original draft, Writing – review & editing.
